# Generation of tissues outside the field of view (FOV) of radiation therapy simulation imaging based on machine learning and patient body outline (PBO)

**DOI:** 10.1186/s13014-023-02384-4

**Published:** 2024-01-25

**Authors:** Sunmi Kim, Lulin Yuan, Siyong Kim, Tae Suk Suh

**Affiliations:** 1 Department of Biomedical Engineering and Research Institute of Biomedical Engineering, College of Medicine, The Catholic University of Korea, 222 Banpo-daero, Seocho−gu, Seoul, 06591 Republic of Korea; 2 Department of Radiation Oncology, School of Medicine, Virginia Commonwealth University, Richmond, VA 23284 USA; 3 Department of Radiation Oncology, Yonsei Cancer Center, Seoul, 03722 Republic of Korea

**Keywords:** Machine learning, Missing tissue generation, Radiation therapy simulation, Field of view (FOV), MR-only simulation

## Abstract

**Background:**

It is not unusual to see some parts of tissues are excluded in the field of view of CT simulation images. A typical mitigation is to avoid beams entering the missing body parts at the cost of sub-optimal planning.

**Methods:**

This study is to solve the problem by developing 3 methods, (1) deep learning (DL) mechanism for missing tissue generation, (2) using patient body outline (PBO) based on surface imaging, and (3) hybrid method combining DL and PBO. The DL model was built upon a Globally and Locally Consistent Image Completion to learn features by Convolutional Neural Networks-based inpainting, based on Generative Adversarial Network. The database used comprised 10,005 CT training slices of 322 lung cancer patients and 166 CT evaluation test slices of 15 patients. CT images were from the publicly available database of the Cancer Imaging Archive. Since existing data were used PBOs were acquired from the CT images. For evaluation, Structural Similarity Index Metric (SSIM), Root Mean Square Error (RMSE) and Peak signal-to-noise ratio (PSNR) were evaluated. For dosimetric validation, dynamic conformal arc plans were made with the ground truth images and images generated by the proposed method. Gamma analysis was conducted at relatively strict criteria of 1%/1 mm (dose difference/distance to agreement) and 2%/2 mm under three dose thresholds of 1%, 10% and 50% of the maximum dose in the plans made on the ground truth image sets.

**Results:**

The average SSIM in generation part only was 0.06 at epoch 100 but reached 0.86 at epoch 1500. Accordingly, the average SSIM in the whole image also improved from 0.86 to 0.97. At epoch 1500, the average values of RMSE and PSNR in the whole image were 7.4 and 30.9, respectively. Gamma analysis showed excellent agreement with the hybrid method (equal to or higher than 96.6% of the mean of pass rates for all scenarios).

**Conclusions:**

It was first demonstrated that missing tissues in simulation imaging could be generated with high similarity, and dosimetric limitation could be overcome. The benefit of this study can be significantly enlarged when MR-only simulation is considered.

## Background

**Fig. 1:**
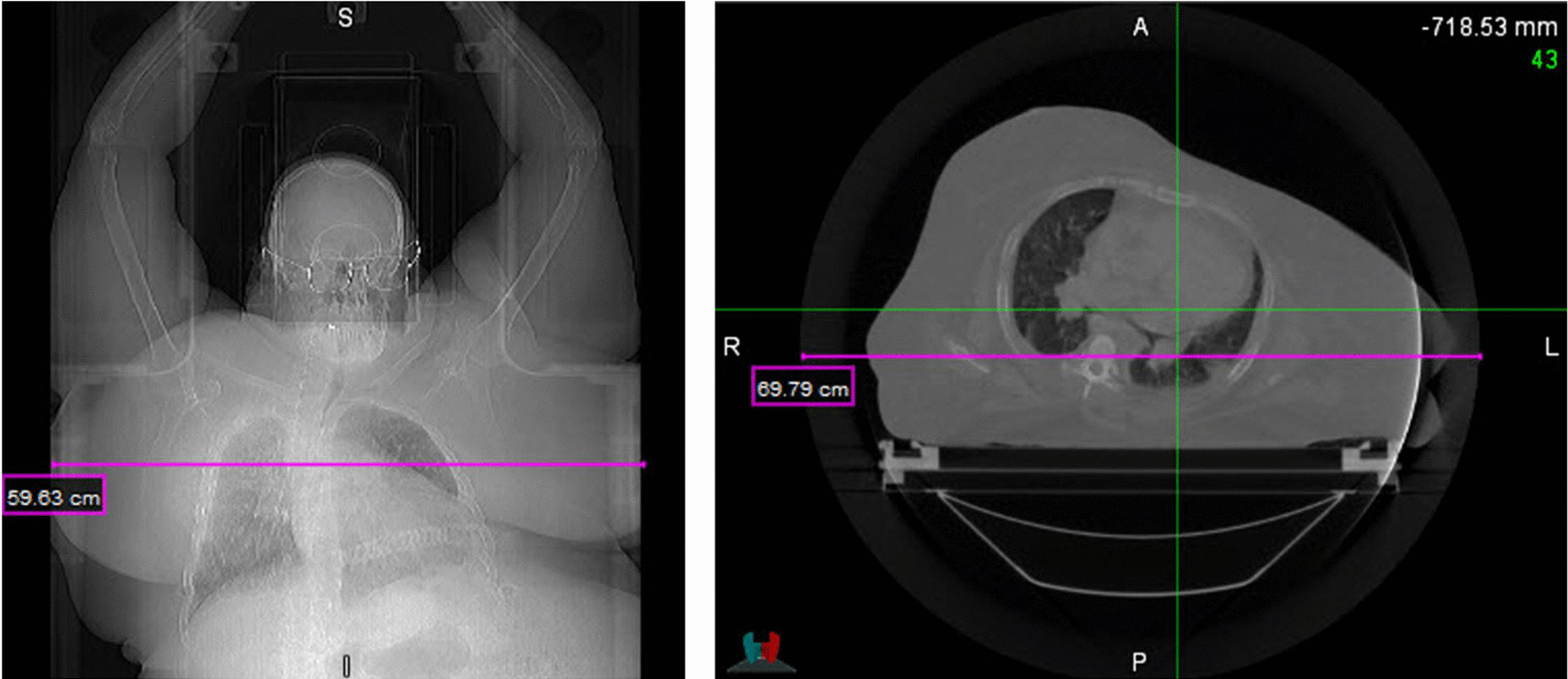
An example of CT image where a part of body was missing even with a 70 cm extended FOV in an 85 cm large bore CT simulator

The image for diagnosis requires accurate and detailed pictures of tumor or the region of interest. Thus, obtaining the whole-body outline in every slice of computed tomography (CT) scanning is not necessary. However, images for treatment plan in radiation therapy are often required to contain the whole body outline for optimal dose planning. Due to the finite size of bore dimension of a CT simulator it is not unusual to encounter a situation where some parts of tissues are not included in the maximum field of view (FOV) of CT simulation images depending on the situation (e.g., when a patient is larger than the maximum FOV or an off-centered setup is needed). Figure 1, for instance, shows a CT image where a part of body was missing even with a 70 cm extended FOV in an 85 cm large bore CT simulator. In such situations, most planners try to make a plan with avoiding beams entering the patient body through the areas of missing tissues, which accordingly hinder the dose planning from being optimally processed, especially when an advanced delivery technique is utilized such as intensity modulated radiation therapy (IMRT) or volumetric modulated arc therapy (VMAT) that typically brings the largest benefit with more flexible beam arrangements in general. Therefore, patients would lose a chance to get an optimal care when missing tissues exist outside the FOV of simulation imaging.

CT has the advantage of offering both accurate surface of patient and CT Values that can be converted to electron densities for radiation dose calculation, which is indispensable for radiotherapy treatment planning. However, CT images often have limited soft tissue contrast, causing difficulty in identifying the tumor and/or adjacent critical structures [1, 2]. On the other hand, magnetic resonance (MR) imaging provides superior soft tissue contrast compared to CT, enabling more accurate delineation of both the target and critical structures [3, 4]. MR is also a multi-parametric imaging modality that can provide not only anatomical information with high soft tissue contrast but also valuable functional information for the assessment of both disease progression and treatment response [5–7]. No imaging dose by ionizing radiation with MRI imaging is another advantage [8].

Radiation treatment planning frequently uses both CT and MR for many disease sites [9, 10]. In general, a planning CT scan is used as the primary image set and MR set is registered as 2nd data set to the CT set. One of the biggest challenges in this approach is the potential systematic error existing in the registration process [11–15]. Obviously, such registration errors can be eliminated through MR based simulation and, in recent years, interests in replacing CT with MR in the treatment planning process have grown rapidly [16–19]. However, most MR units provide a maximum bore size of 70 cm [20, 21] while most CT simulators have an aperture of from 80 to 90 cm. Therefore, the issue of missing tissue due to limited bore size in MR-only simulation can be much more serious.

There have been several papers on how to compensate for data truncation based on Cone Beam CT. [22, 23] However, to our best knowledge, there has been no attempt to solve this issue for radiation treatment planning with initial simulation images. In this study 3 novel methods were proposed to manage missing tissues outside a FOV during simulation imaging without re-simulation. First approach utilizes deep learning (DL) and second does patient body outline (PBO) obtained with an optical surface imaging. Third is based on both DL and PBO combined. In the DL method, missing tissue generation is based on deep convolutional neural network (CNN) and Generative Adversarial Networks (GAN) [24–28]. A proof-of-concept study was performed with a set of CT images.

## Methods

### Imaging data

The imaging database comprised 10,005 CT training slices of 322 lung cancer patients and 166 CT test (i.e., evaluation) slices of 15 lung cancer patients obtained from the publicly available database of the Cancer Imaging Archive (TCIA) [29]. The data are organized as collections. This collection contains images from 422 non-small cell lung cancer (NSCLC) patients. For these patient’s pretreatment CT scans, manual delineation by a radiation oncologist of the 3D volume of the gross tumor volume and clinical outcome data are available. This dataset refers to the Lung1 dataset of the study published in Nature Communications. DICOM is the primary file format used by TCIA for radiology imaging.

Image pre- and post-processing was implemented using MATLAB program and the deep learning network was configured and coded using the Keras package with Tensorflow as the computing backend [30]. To create the cropped CT images to be used as training data, preprocessing was performed using MATLAB. Original CT matrix size was 512 × 512 pixels with 3 mm slice thickness. Using Tensorflow, all of CT images were reduced to 128 × 128 with 256 Gy scale to minimize computational burden in this proof-of-concept simulation.

In this proof-of-concept study we intentionally chose lung patient data. Lung is a body site where large uncertainty is expected when missing parts are generated due to significant density variaton. Therefore, lung is considered one of the most challenging body sites to demonstrate that the proposed method works.

### Architecture for missing tissue generation in deep learning (DL) method

In this section, we explain the process of generating CT missing tissue using deep learning. In the early stages of the research, there was an experiment process comparing different architectures [31–36] and we chose Globally and Locally Consistent Image Completion (GLCIC) as an optimal one for this study. Compared to other networks, GLCIC was superior in terms of image size and shape, resolution of generated image, and computation time. Our work builds upon a Globally and Locally Consistent Image Completion (GLCIC) [35] to learn features by Convolutional Neural Networks-based (CNN-based) inpainting, based on Generative Adversarial Network (GAN).

More specifically, The GLCIC consists of three networks, a completion network, the global context discriminator, and the local context discriminator [35]. A unique feature of GLCIC network structure is that a dilated convolution layer [30, 31, 37, 38] is used for the completion network rather than a general convolution layer. Dilated convolution has the same number of parameters and amount of computation as those of general convolution but has the advantage of being able to view a much wider area at once. Using dilated convolution, it allows us to understand the context of an image without using fully connected layers, hence the trained network can be used for images with diverse sizes. Next, it is divided into global and local discriminator, and inserts the whole image and blank part respectively. Two discriminators ensure the global and local consistency of the filled images. The global discriminator takes the whole image as input to recognize global consistency of the image while the local discriminator focuses on a small region (generated region). The main role of discriminators is to determine whether an image has been completed consistently. Also, It is the importance of generating novel fragments in the task of image inpainting. We adopt pixel-wise reconstruction loss (L2 loss) to ensure that we can fill in the missing parts with “correct” structure.

**Fig. 2:**
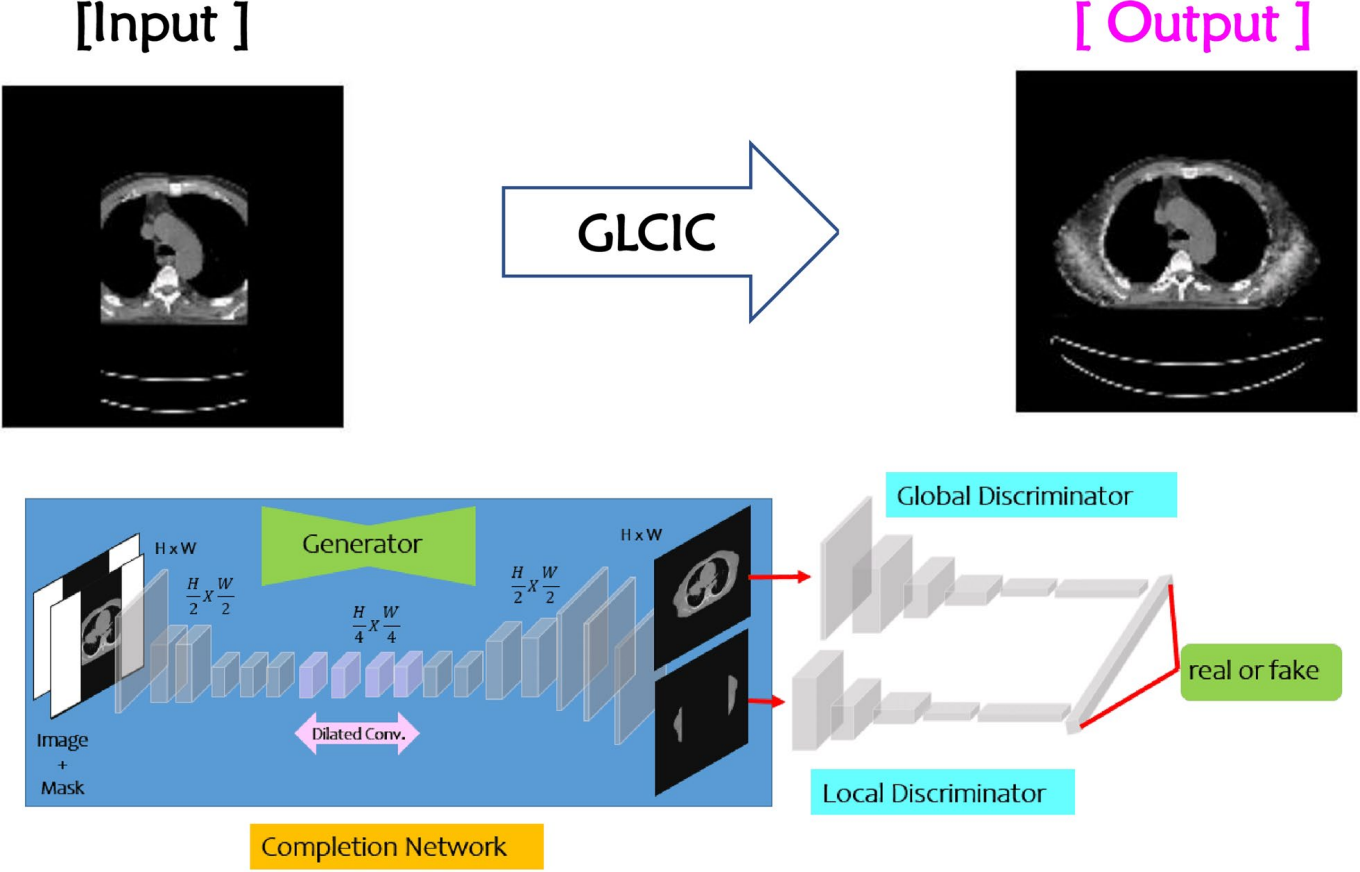
Overall scheme of Globally and Locally Consistent Image Completion (GLCIC) Network for missing tissue generation

Figure 2 illustrates the overall scheme of the process. A CT image with missing parts (input) is expanded to a CT image with the missing parts generated (output) using a machine learning based algorithm. To make an input image a total of 72 × 128 pixels (i.e., about 56% of the original image) were replaced with “0” (i.e., 36 × 128 from the left side and another 36 × 128 from the right) as shown in the top left image of Fig. 2.

During each training iteration, the discriminators are updated first so that they correctly distinguish between real and completed training images. Afterwards, the completion network is updated so that it fills the missing area well enough to fool the context discriminator networks. Using both the local and the global context discriminators is helpful for obtaining overall image completion. In contrast to patch-based approaches like PatchMatch [39, 40] GLCIC approach can generate novel fragments, which allows completing the images of objects with highly specific structures.

The architecture of the image completion network is provided in Table 1. After each convolution layer, except the last one, there is a Rectified Linear Unit (ReLU) layer. The output layer consists of a convolutional layer with a sigmoid function instead of a ReLU layer to normalize the output to the [0, 1] range. “Outputs” refers to the number of output channels for the output of the layer.

**Table 1:** Architecture of the image completion network

Type	Kernel	Dilation(η)	Stride	Outputs
Conv	5 × 5	1	1 × 1	64
Conv	3 × 3	1	2 × 2	128
Conv	3 × 3	1	1 × 1	128
Conv	3 × 3	1	2 × 2	256
Conv	3 × 3	1	1 × 1	256
Conv	3 × 3	1	1 × 1	256
Dilated conv	3 × 3	2	1 × 1	256
Dilated conv	3 × 3	4	1 × 1	256
Dilated conv	3 × 3	8	1 × 1	256
Dilated conv	3 × 3	16	1 × 1	256
Conv	3 × 3	1	1 × 1	256
Conv	3 × 3	1	1 × 1	256
Deconv	4 × 4	1	1/2 × 1/2	128
Conv	3 × 3	1	1 × 1	128
Deconv	4 × 4	1	1/2 × 1/2	64
Conv	3 × 3	1	1 × 1	32
Output	3 × 3	1	1 × 1	3

Conv., Convolution layer; Dilated conv., Dilated convolution layer; Deconv., Deconvolution layer

Table 2. summarizes the architectures of the discriminators used. As seen in standard neural networks, Full Connected (FC) layers can be used to optimize objectives. In this study, the output layer consists of a FC layer with a sigmoid transfer function.

**Table 2:** Architectures of the discriminators used in the network model: (a) Local Discriminator, (b) Global Discriminator, and (c) Concatenation Layer

Type	Kernel	Stride	Outputs
(a) Local Discriminator			
Conv	5 × 5	2 × 2	64
Conv	5 × 5	2 × 2	128
Conv	5 × 5	2 × 2	256
Conv	5 × 5	2 × 2	512
Conv	5 × 5	2 × 2	512
FC	–	–	1024

Type	Kernel	Stride	Outputs
(b) Global Discriminator			
Conv	5 × 5	2 × 2	64
Conv	5 × 5	2 × 2	128
Conv	5 × 5	2 × 2	256
Conv	5 × 5	2 × 2	512
Conv	5 × 5	2 × 2	512
Conv	5 × 5	2 × 2	512
FC	–	–	1024

Type	Kernel	Stride	Outputs
(c) Concatenation Layer			
Conv	–	–	2048
FC	–	–	1

Conv., Convolution layer; FC, Fully-connected layer

### Patient body outline (PBO) method

Second approach is to obtain a PBO in interest. PBOs will be obtained using an optical surface imaging in actual practice as illustrated in a study performed by our group [41] where, it was demonstrated that a whole body image set for a total body irradiation (TBI) treatment planning could be obtained in a single setup by using both CT and 3D surface imaging. Note surface imaging methods can provide large FOVs easily by either having multiple cameras or rotating a camera.

However, in this simulation study with exsisting archived CT data the PBOs have been simply obtained from the original CT slices. Missing tissue parts are simply filled with water density.

### Hybrid method combining DL and PBO

**Fig. 3:**
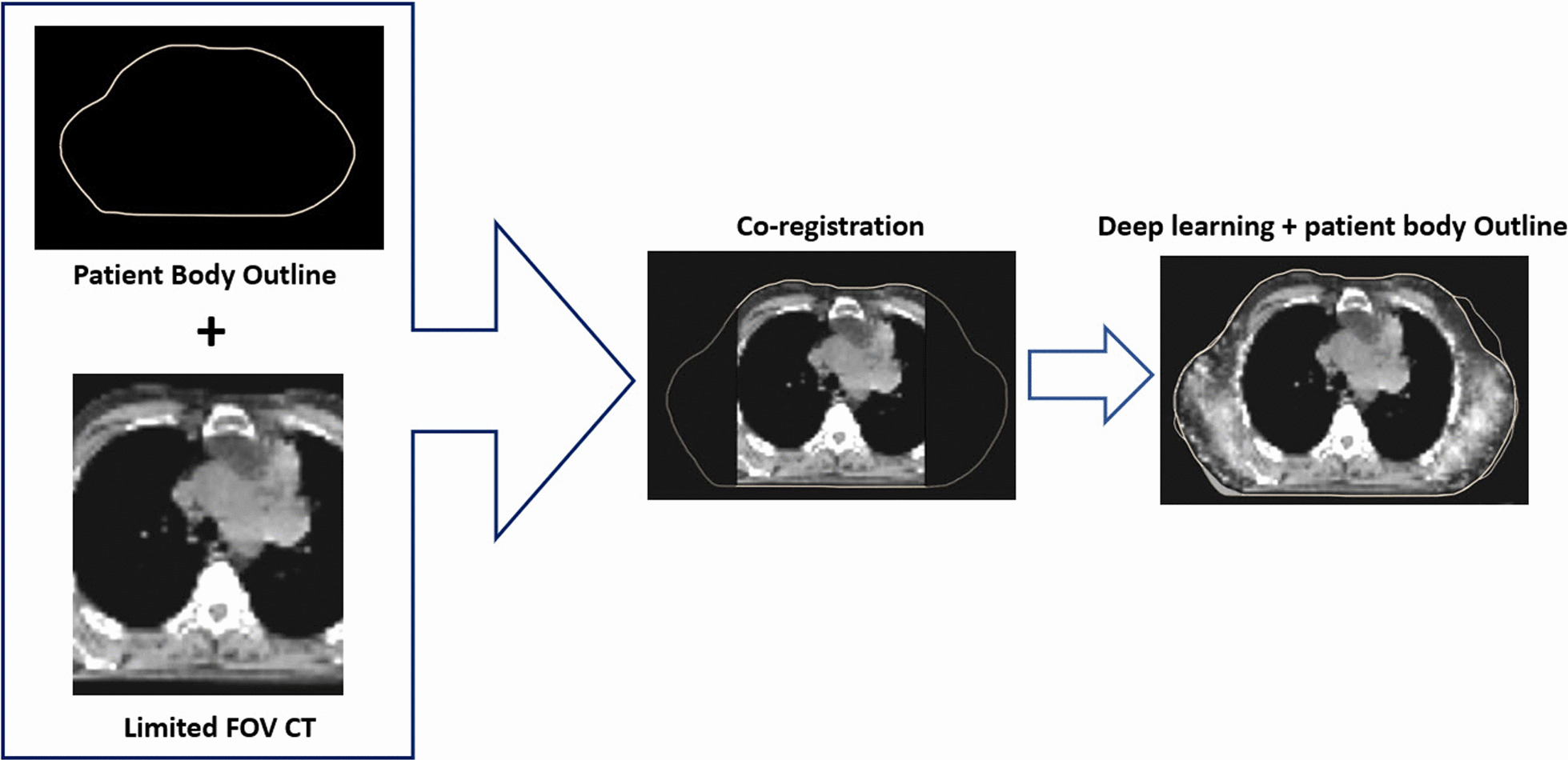
Fine-tuning of machine learning generated image based on PBO

The generated missing tissues by the machine learning in Sect. "Architecture for missing tissue generation in deep learning (DL) method" are fine-tuned using the PBOs. In specific, if there exist artificially generated tissues outside the PBOs they are eliminated. When there are still missing tissues inside the PBOs they are replaced with water. This process is illustrated in Fig. 3.

### Image evaluation metrics

For quantitative assessment of the performance level of the process, Root Mean Square Error (RMSE) [42], Peak signal-to-noise ratio (PSNR) and Structural Similarity Index Metric (SSIM) [43, 44] were evaluated. RMSE is a type of error measuring techniques commonly used to measure the differences between the predicted value by an estimator and the actual value. PSNR is the ratio of the maximum possible signal power and the corrupting noise power. The PSNR calculates the PSNR ratio in decibels amid two images. SSIM is a full reference metric. It compares two images using information about luminance, contrast and structure.

Mean Square Error (MSE), RMSE, PSNR, and SSIM are defined in formulas (1), (2), (3) and (4), respectively.

1 $$MSE=\frac{1}{n}\sum_{i=1}^{n}{\left({\widehat{y}}_{i}-{y}_{i}\right)}^{2}$$

2 $$RMSE=\sqrt{MSE}$$

3 $$PSNR=10\mathit{log}\frac{{S}^{2}}{MSE}$$

where, s is the maximum possible pixel value of the image. When the pixels are represented using 8 bits per sample, it is supposed to be 255.

4 $$SSIM\left(x,y\right)=\frac{\left(2{\mu }_{x}{\mu }_{y}+{C}_{1}\right)\left(2{\sigma }_{xy}+{C}_{2}\right)}{\left({\mu }_{x}^{2}+{\mu }_{y}^{2}+{C}_{1}\right)\left({\sigma }_{x}^{2}+{\sigma }_{y}^{2}+{C}_{2}\right)}$$

where,

${\widehat{y}}_{i}$: predicted output value.

${y}_{i}$: actual output value.

${\upmu }_{\mathrm{x}}$: the average of x.

${\upmu }_{\mathrm{y}}$: the average of y.

${\upsigma }_{\mathrm{x}}^{2}$: the variance of x.

${\upsigma }_{\mathrm{y}}^{2}$: the variance of y.

${\upsigma }_{\mathrm{xy}}$: the covariance of x and y.

${\mathrm{C}}_{1}={\left({\mathrm{k}}_{1}\mathrm{L}\right)}^{2}$, ${\mathrm{C}}_{2}={\left({\mathrm{k}}_{2}\mathrm{L}\right)}^{2}$: two variables to stabilize the division with weak denominator.

L: the dynamic range of the pixel-values.

k_1_ = 0.01 and k_2_ = 0.03 by default.

### Dosimetric evaluation

For dosimetric validation a dynamic conformal arc (DCA) plan was considered since it often requires a full body contour for adequate optimization. For each test case, a DCA plan was made with the ground truth image set first, the same plan was applied to the image sets obtained by 3 proposed methods, and then calculated doses were compared in terms of gamma-evaluation. In this simulation, a 2 cm diametter sherical target with 200 cGy prescribed dose was used. Eclipse Acuros XB advanced dose calculation algorithm was used for dose calculation. Gamma analysis was conducted at relatively strict criteria of 1%/1 mm (dose difference/distance to agreement) and 2%/2 mm under three dose thresholds of 1%, 10% and 50% of the maximum dose in the plans made on the ground truth image sets.

## Results

### Training loss in DL

**Fig. 4:**
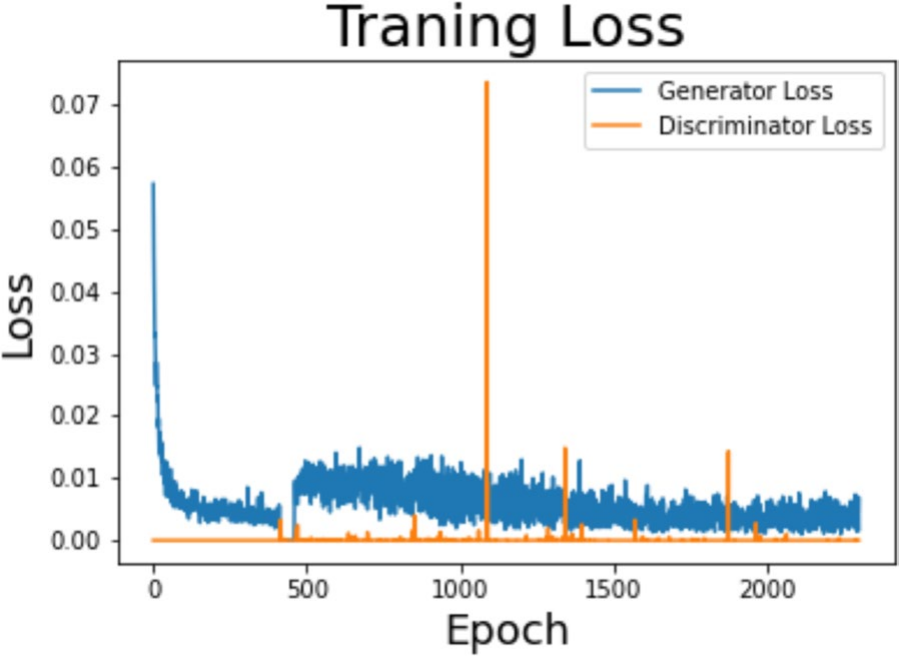
Training generator loss and discriminator loss over 2300 epochs: Note the generator tries to minimize the loss while the discriminator does to maximize it

The standard GAN loss function, described in the 2014 GAN paper by Ian Goodfellow et al. [25], is also known as the min–max loss. It can further be categorized into two parts: Discriminator loss and Generator loss. The generator tries to minimize the loss while the discriminator does to maximize it. In practice, it saturates for the generator, meaning that the generator quite frequently stops training if it does not catch up with the discriminator. Figure 4 displays the plots of training generator loss and discriminator loss according to epoch up to 2,300. Generator and discriminator are roughly balanced, but discriminator is more consistent. Occasional "spikes" come along associated with very high gradient norms. These come with dramatic updates to generator.

### Qualitative evaluation in DL

**Fig. 5:**
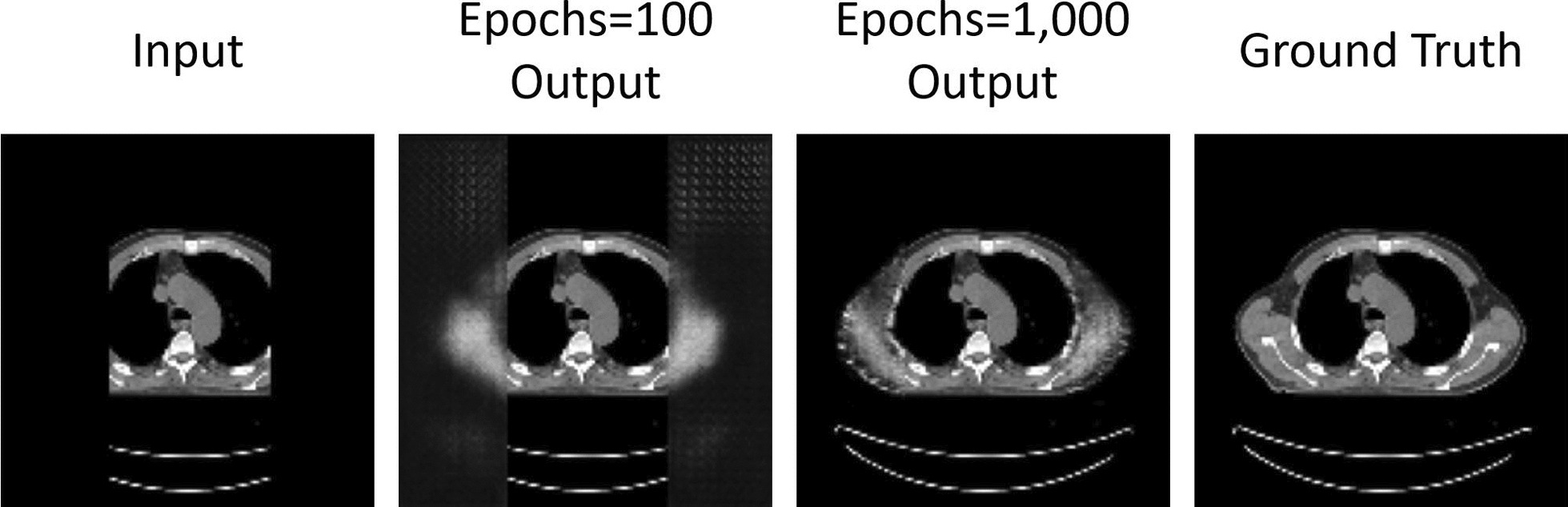
Illustration of the outputs of test image of case#1 obtained by the model according to the number of epochs. The input image size is 128 × 128 with the batch size of 2. From left to right, displayed are input images, outputs at epochs of 100 and 1,000, and ground truth images

Figure 5 illustrates the outputs of 2 test images, obtained by the model developed, according to the number of epochs. The left most imgeas are inputs and the right most ones are ground truth images. In between, output images are displayed at 2 epochs, 100 and 1000.

As can be seen missing tissues were generated and the output iamges got more similar to the ground truth images with the number of epochs increased within the range shown.

### Quantitative evaluation in DL

**Fig. 6:**
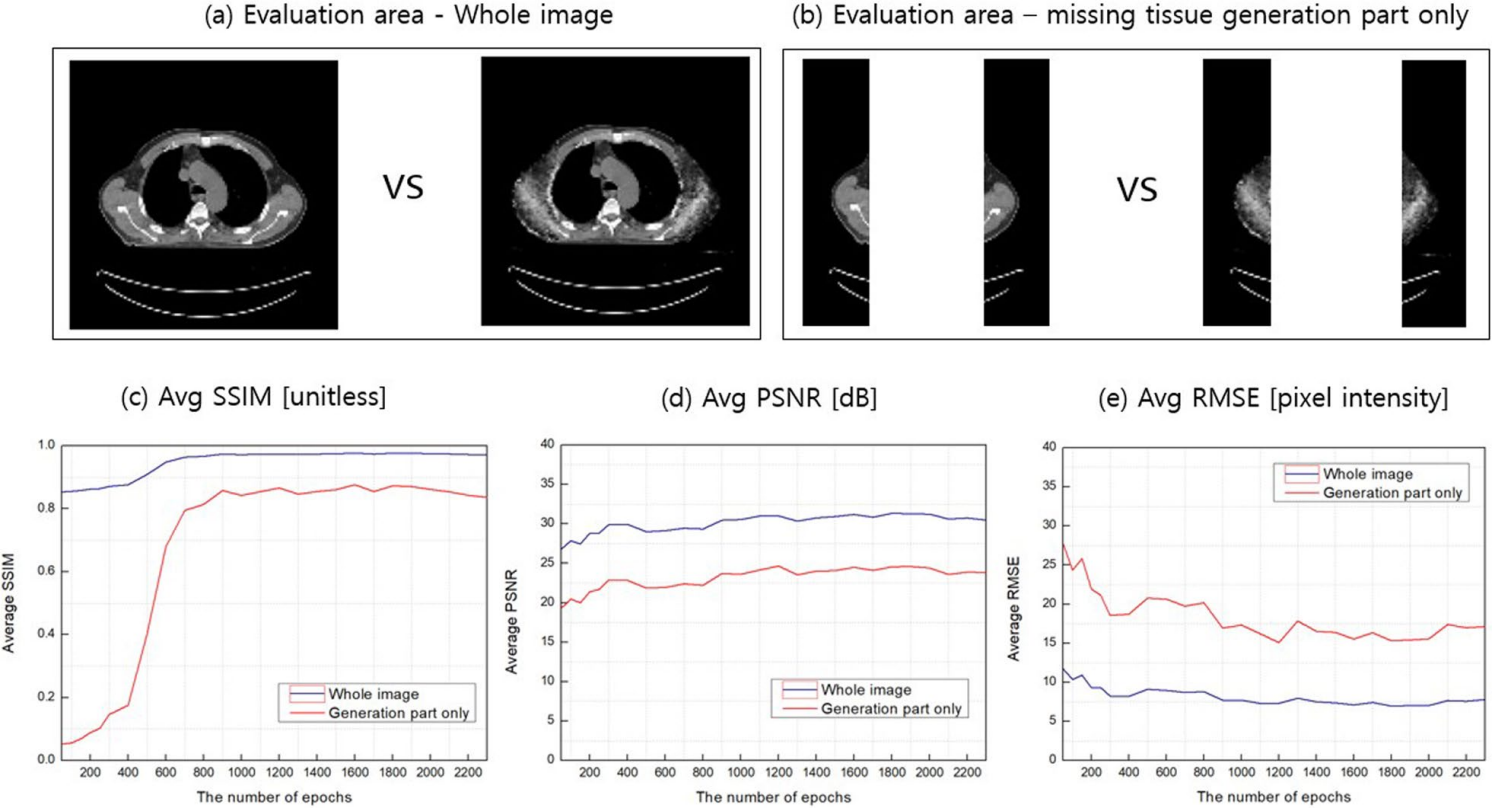
Quantitative evaluation results with the number of epochs: **a** Evaluation area – whole image, **b** Evaluation area – missing tissue generation part only **c** Average structural similarity index (SSIM), **d** Average peak signal-to-nose ratio (PSNR) and **e**, Average root mean square error (RMSE)

The quantitative metrics, SSIM, PSNR and RMSE averaged over whole test set (i.e., 166 slices) are plotted according to the number of epochs in Fig. 6. Values were calculated in 2 different areas, the whole area as illustrated in Fig. 6a and missing tissue generation part only as shown in Fig. 6b. The red lines are for the latter case and the black the former. While the evaluation on the missing tissue generation area only was our main interest in this study, that on the whole area of image would provide overall similarity. As can be seen in Fig. 6c, the average SSIM in missing tissue generation area significantly increased with the number of epochs, for example, it was 0.06 at epoch 100 but reached to 0.86 at epoch 1500. Accordingly, the average SSIM in the whole area of the image also increased from 0.86 to 0.97.

The average PSNR, as displayed in Fig. 6d, increased from around 20 and 28 to 24 and 31 for the missing tissue generation area only and the whole area, respectively.

On the other hand, the average RMSE decreased from around 25 and 10 to 15 and 7 for the missing tissue generation area only and the whole area, respectively, as shown in Fig. 6e. Numerical average values of SSIM, RMSE and PSNR are summarized in Table 3 for 5 epochs (100, 500, 1000, 1500 and 2000).

**Table 3:** The average SSIM, PSNR and RMSE values according to the number of epochs

Epoch	Structural Similarity Index (SSIM) between output and ground truth	
	Generation part only	Whole image
0	–	0.9434
100	0.0557	0.8552
500	0.4004	0.9086
1,000	0.8421	0.9715
1,500	0.8603	0.9745
2,000	0.8613	0.9747

Epoch	Peak signal-to-noise ratio (PSNR) between output and ground truth	
	Generation part only	Whole image
0	–	22.0114
100	20.4575	27.8581
500	21.8561	28.9943
1,000	23.6109	30.5355
1,500	24.0551	30.9155
2,000	24.3891	31.2220

Epoch	Root Mean Square Error (RMSE) between output and ground truth	
	Generation part only	Whole image
0	–	20.2287
100	24.3296	10.3563
500	20.7680	9.0974
1000	17.3119	7.7092
1500	16.3794	7.3581
2000	15.5609	7.0198

### Dosimetric evaluation

The gamma analysis under the 1%/1 mm and 2%/2 mm criteria for 15 cases are summarized in Table 4. As shown in the table the best results were obtained with the proposed hybrid method (i.e., DL + PBO approach) among 3 approaches proposed. In detail, the mean of pass rates under all thresholds considered were equal to or higher than 96.6% and 99.2% for 1%/1 mm and 2%/2 mm criterion, respectively. In high dose region (i.e., under 50% threshold) the pass rates of hybrid method were 100% for all cases except one which was 99.3% for case 11 with 1%/1 mm criterion. The DL only approach provided good agreements with the mean of pass rates ranged from ~ 93% to ~ 100% among the thresholds considered. The results of PBO only method were the worst with the mean of pass rates ranging from ~ 86% to ~ 98% among the thresholds considered.

**Table 4:** Gamma Analysis Results: all of pass rates of 100% are highlighted in bold to be emphasized; note, for the hybrid (i.e., DL + PBO) method, pass rates under 50% threshold were 100% for all cases except one which was 99.3% for case 11 with 1%/1 mm criterion

	1%/1 mm									2%/2 mm								
	DL only			PBO only			DL + PBO			DL only			PBO only			DL + PBO		
	Threshold			Threshold			Threshold			Threshold			Threshold			Threshold		
Case#	1%	10%	50%	1%	10%	50%	1%	10%	50%	1%	10%	50%	1%	10%	50%	1%	10%	50%
1	95.4	95.9	**100.0**	95.4	89.6	89.3	99.3	97.5	**100.0**	99.6	99.7	**100.0**	98.7	95.7	96.7	**100.0**	99.9	**100.0**
2	96.5	95.8	**100.0**	90.4	79.1	56.5	99.1	97.3	**100.0**	98.8	99.1	**100.0**	95.6	88.9	72.1	99.9	99.5	**100.0**
3	96.3	99.7	**100.0**	98.0	95.8	**100.0**	**100.0**	**100.0**	**100.0**	98.9	100.0	**100.0**	99.9	99.8	**100.0**	**100.0**	**100.0**	**100.0**
4	95.4	96.3	99.2	91.7	82.3	90.8	99.2	97.3	**100.0**	98.9	98.6	**100.0**	96.9	90.2	**100.0**	99.7	98.7	**100.0**
5	93.1	92.3	**100.0**	94.4	87.3	**100.0**	96.9	92.3	**100.0**	97.0	96.0	**100.0**	98.5	95.0	**100.0**	98.8	96.7	**100.0**
6	95.0	95.9	**100.0**	96.3	89.9	**100.0**	99.5	99.1	**100.0**	98.9	99.8	**100.0**	99.1	96.8	**100.0**	**100.0**	99.9	**100.0**
7	95.0	94.3	97.5	92.5	86.9	80.7	98.8	96.0	**100.0**	98.4	98.6	**100.0**	97.1	92.8	99.7	99.8	99.2	**100.0**
8	92.6	95.9	**100.0**	92.1	88.3	**100.0**	98.8	97.8	**100.0**	96.8	99.0	**100.0**	98.3	95.3	**100.0**	**100.0**	99.9	**100.0**
9	96.0	97.1	98.3	96.5	94.7	**100.0**	99.7	99.0	**100.0**	99.2	99.9	**100.0**	99.1	97.9	**100.0**	**100.0**	**100.0**	**100.0**
10	94.3	94.7	**100.0**	92.8	87.1	79.0	99.2	97.5	**100.0**	97.8	98.5	**100.0**	97.0	92.5	95.6	99.9	99.4	**100.0**
11	85.7	85.5	98.7	86.9	69.8	81.3	95.0	89.8	99.3	94.3	95.3	**100.0**	95.3	84.0	93.9	99.2	97.1	**100.0**
12	88.9	92.6	99.4	94.0	92.2	**100.0**	95.1	94.7	**100.0**	93.9	96.8	**100.0**	99.2	98.2	**100.0**	99.0	98.7	**100.0**
13	95.3	95.4	**100.0**	99.3	98.1	**100.0**	99.7	99.3	**100.0**	99.0	98.9	**100.0**	99.9	99.8	**100.0**	**100.0**	99.8	**100.0**
14	93.1	95.3	**100.0**	93.3	87.7	**100.0**	98.8	97.4	**100.0**	98.0	99.1	**100.0**	98.5	95.6	**100.0**	**100.0**	**100.0**	**100.0**
15	90.9	88.4	99.3	82.3	66.9	24.0	98.3	94.8	**100.0**	96.8	97.2	**100.0**	92.0	79.6	33.7	99.9	99.6	**100.0**
*Mean*	*93.6*	*94.3*	*99.5*	*93.1*	*86.4*	*86.8*	*98.5*	*96.6*	*100.0*	*97.7*	*98.4*	*100.0*	*97.7*	*93.5*	*92.8*	*99.7*	*99.2*	*100.0*
*SD*	*2.9*	*3.4*	*0.8*	*4.1*	*8.5*	*20.7*	*1.5*	*2.7*	*0.2*	*1.7*	*1.4*	*0.0*	*2.1*	*5.5*	*17.2*	*0.4*	*1.0*	*0.0*

**Fig. 7:**
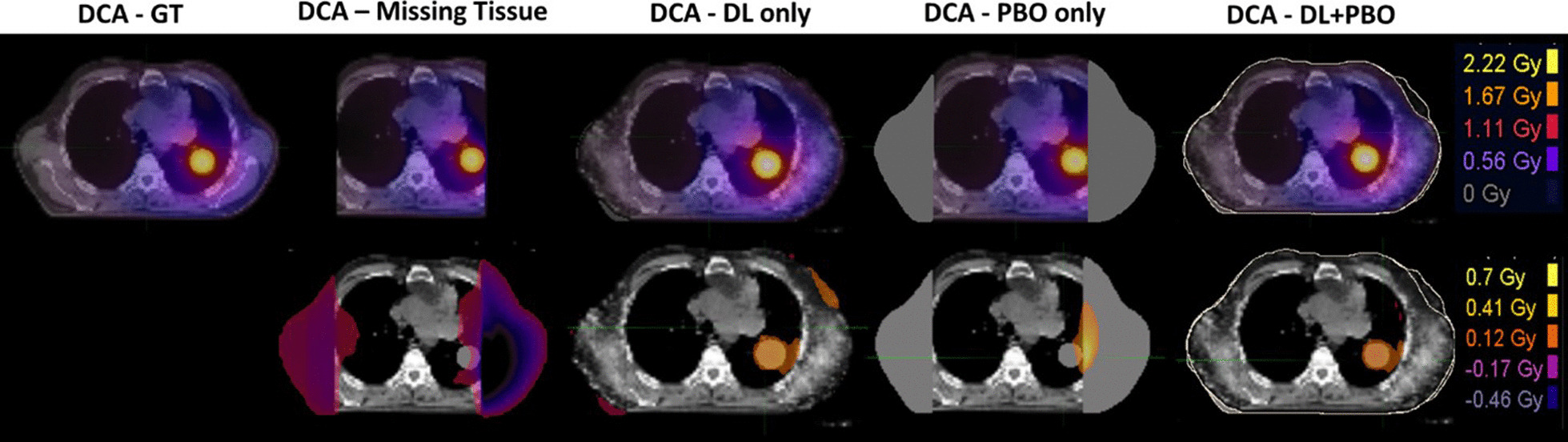
An illustration of dose distributions of the case 2. The first row are the ground truth (GT) and calculated dose distributions; the second row are the dose difference map between the calculated and GT dose. Dose differences less than 0.1 Gy are not shown in the figures. In columns from left to right, plans on GT image set, GT image with missing tissue removed, deep learning (DL) only image set, patient body outline (PBO) only image set and DL + PBO image set

Figure 7 shows dose distributions of the case 2 as an illustration.

### Computation time

The weighting hyper parameter was set to α = 0.0004. The batch size of 2 was used for training. The completion network was trained up to 2300 iterations. The entire training procedure took roughly 11 days on one workstation that included an NVIDIA GeForce GTX 970 graphic Card. The training of the GLCIC per epoch took approximately 7 min. When the model is trained, it takes approximately 0.5 s for missing tissue generation.

## Discussion

Recently, convolutional neural networks (CNN) based deep learning has been utilized to medical imaging with successful implementations for a wide range of applications [45–47]. A study in Ref. Han [48] demonstrated the feasibility of pseudo CT generation from MR images with a deep CNN model. This has led to the development of several approaches for the generation and translation of image data. Our study described in this article aimed to investigate whether machine learning approach could generate missing body parts in medical imaging and demonstrated it was possible using a set of CT images, for the first time to our best knowledge.

For evaluating the performance of the DL-generated images, Structural Similarity Index Metric (SSIM), Root Mean Square Error (RMSE) and Peak signal-to-noise ratio (PSNR) were evaluated [49, 50]. SSIM is based on visible structures in the image thus, it actually measures the perceptual difference between two similar images. An SSIM value is between 0 and 1 with 1 indicating perfect structural similarity. Therefore, it is considered the model is able to generate images in high similarity. This consideration is also supported by both RMSE and PSNR values. RMSE measures the amount of change per pixel due to a process. RMSE values are non-negative and a value of 0 means the image being compared are identical. PSNR measures the ratio between the maximum possible power of a signal and the power of corrupting noise that affects the fidelity of its representation. PSNR is usually expressed in terms of the logarithmic decibel scale. A 20 dB or higher PSNR indicates that the image is of good quality. Obviously, a smaller value of RMSE and a higher value of PSNR indicate that the images are of higher quality. In quantitative analysis, the output images showed the average SSIM values of up to 0.86 and 0.97 for the generated part only and the whole area, respectively. Also, when epoch is 1500, average values of RMSE and PSNR in the whole image are 7.4 and 30.9, respectively. Even in the missing tissue generation area only, PSNR is 24.1 which is larger than 20 with enough margin.

Although this study did not specifically address how to obtain 3D surface imaging, our team members have developed a technology that utilizes both CT and 3D optical surface imaging to acquire 3D whole-body information and import it into a treatment planning system (TPS) for total body irradiation planning [41]. Nevertheless, surface imaging of posterior parts of the body can be challenging. However, we believe, PBO can be obtained with acceptable accuracy in most cases. For most patients, the outline under the body (i.e., posterior part of the body) is typically inside the FOV thus only lateral parts need to be included in surface imaging. For obese patients, both lateral and laterally located posterior parts of the body would be missing. In this case, lateral parts can be surfaced imaged but laterally located posterior parts can be estimated to be flat (due to the table) or approximated with the surface of the immobilization device if immobilized.

The MRI-only treatment process is currently an active field of research since it could eliminate systematic MR-CT co-registration errors [13–15], reduce medical cost, avoid diagnostic radiation exposure, and simplify clinical workflow. However, most MR bores are smaller than those of CT, resulting in higher chances of having missing tissues outside the FOV [20]. When an MRI system is used for simulation in radiation therapy, a synthetic CT is generated for both dose calculation and reference image preparation. While MRI data set provides superior soft tissue contrast over that of CT, one of distinct limitations of MRI system at present is its smaller field of view (FOV), resulting in exclusion of patients in relatively large size. To solve the issue of small MR FOV, a process of creating tissue information not included in the FOV is under consideration. In step 1, patient body outline (3D surface imaging) in interest is obtained using an optical imaging method. In step 2, an MR image set in limited FOV is converted to a synthetic CT image set in limited FOV. In step 3, the syn-CT-in-LFOV is expanded to a synthetic CT image set in full FOV using both the patient body outline obtained in the step 1 and a machine learning based missing body generation algorithm. This approach is also able to significantly reduce geometrical distortion that is dominant at periphery in typical MR images. In this study, we focused on Step 3 as a novel development. For Step 1 and Step 2, there are already promising technologies available as mentioned above. When all three steps are integrated, we would be able to solve the issue of small FOV in MR based treatment planning. Therefore, the value of the solution developed in this study can be significantly enlarged in MR-only simulation environment, which is considered one of future directions in radiation oncology practice.

Furthermore, obesity is continuously increasing in the United States such that currently more than 65% of U.S adults are considered overweight or obese and this represents a 25% increase in the past three decades [51–54]. As is to be expected with the increased prevalence of obesity in the general population, the number of obese patients requiring medical imaging also has increased. Therefore, it is important that medical images provide some practical solutions to improve the accuracy of treatment planning imaging in this obese patient population. This study, we believe, can certainly mitigate such problem significantly.

Due to limited computer memory, the network model in this study was designed to take a 2D slice as input and outputs a corresponding 2D slice with the generated missing tissues. In addition, the 2D procedure is still much more efficient than voxel-by-voxel predictions. If necessary, this method can later be extended to take multiple slices as input or process a 3D volume when data and computer resources become available.

We have utilized just one architecture in this study. Since many different network models have been proposed in the CNN algorithms and applications, various architectures can be tested in the future to build a model with improved performance.

While the deep learning method was able to provide missing tissues with decent levels of similarity, obviously, it is hard to expect it can predict exact body outlines. As demonstrated modern surface imaging technologies can be utilized to compensate such limitation. When naked skin surface is not easily obtainable due to practical reasons like cultural barrier thermal imaging technique can be considered. Another potential advantage of surface imaging is that it may be able to reduce image distortion problem during MR-only simulation since distortion is more dominant at body periphery in MR images.

## Conclusions

It was first demonstrated that missing tissues in simulation imaging could be generated with high similarity (reaching up to 0.86 of similarity index) using the machine learning method for all cases tested. Addition of patient body outline information further improved the dosimetric accuracy with the mean of gamma pass rates equal to or higher than 96.6% in all evaluated cases.

## Data Availability

The datasets used and/or analysed during the current study are available from the corresponding author on reasonable request.

## Abbreviations

CT: Computer tomography
FOV: Field of view
IMRT: Intensity modulated radiation therapy
VMAT: Volumetric modulated arc therapy
MR: Magnetic resonance
DL: Deep learning
PBO: Patient body outline
CNN: Convolutional neural network
GAN: Generative Adversarial Networks
TCIA: The Cancer Imaging Archive
GLCIC: Globally and Locally Consistent Image Completion
FC: Fully connected
ReLU: Rectified Linear Unit
Conv.: Convolution layer
Dilated conv.: Dilated convolution layer
Deconv.: Deconvolution layer
TBI: Total body irradiation
RMSE: Root Mean Square Error
PSNR: Peak signal-to-noise ratio
SSIM: Structural Similarity Index Metric
MSE: Mean Square Error
DCA: Dynamic conformal arc
